# Neuroprotective Effects of Choline Alfoscerate in Experimental Diabetic Peripheral Neuropathy

**DOI:** 10.3390/ph19071076

**Published:** 2026-07-12

**Authors:** Hyeri Lee, Hye Won Park, Hyung-Gun Kim, So Hee Hyun, Namhyun Chung, Woon Kyu Lee

**Affiliations:** 1 Department of Biosystems Engineering, College of Life Sciences and Biotechnology, Korea University, Seoul 02841, Republic of Korea; hyeri730@gmail.com (H.L.); nchung@korea.ac.kr (N.C.); 2 Department of Biomedical Sciences, School of Medicine, Inha University, Incheon 22212, Republic of Korea; phw@inha.edu; 3 Biomedical Research Institute, H+ Yangji General Hospital, Seoul 08779, Republic of Korea; m5372@newyih.com; 4 Institute for Clinical and Translational Research, Center for the Improvement of Mentored Experiences in Research, University of Wisconsin-Madison, Madison, WI 53705, USA; hyunsohee07@gmail.com

**Keywords:** diabetic peripheral neuropathy, choline alfoscerate, streptozotocin, neuropathic pain, neuroprotection, triglycerides, sciatic nerve

## Abstract

**Background/Objectives:** Diabetic peripheral neuropathy (DPN) is a common and debilitating complication of diabetes mellitus characterized by progressive nerve degeneration and chronic neuropathic pain. Current therapies, including pregabalin, primarily provide symptomatic pain relief and have limited effects on preventing structural nerve damage. Therefore, the development of disease-modifying therapies remains an important unmet clinical need. This study investigated the neuroprotective effects of choline alfoscerate (CA) and its ability to attenuate mechanical hypersensitivity in a streptozotocin (STZ)-induced rat model of DPN. **Methods:** Diabetes was induced in rats using STZ, and administration protocols were optimized to establish sustained hyperglycemia while minimizing mortality. CA treatment was initiated immediately after STZ administration and continued throughout the study period. Mechanical sensitivity was assessed using the von Frey test. Histopathological examination of sciatic nerves was performed to evaluate structural alterations, and serum biochemical and lipid parameters were analyzed to assess systemic metabolic changes. **Results:** STZ-treated diabetic rats developed persistent hyperglycemia, mechanical allodynia, elevated serum triglyceride levels, and marked structural deterioration of sciatic nerve fascicles. CA treatment significantly increased paw withdrawal thresholds despite sustained hyperglycemia, indicating attenuation of mechanical hypersensitivity independent of glycemic control. Histopathological evaluation demonstrated reduced nerve fiber degeneration, attenuation of edema-like changes, and preservation of sciatic nerve architecture in CA-treated animals. In addition, CA significantly reduced serum triglyceride levels compared with diabetic controls. **Conclusions:** CA attenuated mechanical hypersensitivity and exerted neuroprotective effects in STZ-induced diabetic rats. These benefits occurred independently of glucose lowering and were accompanied by improvements in nerve morphology and lipid metabolism. The findings suggest that CA may represent a promising therapeutic candidate for preserving peripheral nerve integrity and attenuating neuropathic progression in diabetic peripheral neuropathy.

## 1. Introduction

Diabetic peripheral neuropathy (DPN) is among the most prevalent chronic complications of diabetes mellitus and affects approximately 30–50% of individuals with diabetes during their lifetime [1]. As the global prevalence of diabetes continues to increase, the burden of DPN is expected to rise substantially, making it a major cause of disability, chronic pain, foot ulceration, and lower-extremity amputation [2]. DPN is characterized by progressive sensory dysfunction, neuropathic pain, axonal degeneration, and demyelination, resulting in significant impairment of quality of life and increased healthcare burden [3]. Persistent hyperglycemia initiates a complex cascade of metabolic and vascular abnormalities, including oxidative stress, mitochondrial dysfunction, advanced glycation end-product accumulation, and microvascular impairment, all of which contribute to peripheral nerve injury [4].

Current pharmacological management of diabetic peripheral neuropathy (DPN) primarily focuses on symptomatic pain control. Pregabalin (Sigma-Aldrich, St. Louis, MO, USA) and other gabapentinoids are widely prescribed to alleviate neuropathic pain by modulating voltage-gated calcium channels and reducing excitatory neurotransmitter release [5]. Although these agents effectively reduce pain perception, they do not directly prevent axonal degeneration or restore damaged nerve structures. Consequently, increasing attention has been directed toward the development of disease-modifying therapies that target the underlying pathogenic mechanisms responsible for neuropathic progression [6]. Furthermore, long-term use of these agents is frequently associated with adverse effects such as dizziness, somnolence, peripheral edema, and cognitive impairment, highlighting the need for alternative therapeutic strategies capable of providing both symptomatic relief and protection against progressive nerve damage [7].

Recent evidence suggests that disturbances in lipid metabolism play a critical role in the pathogenesis of diabetic peripheral neuropathy [8]. Hypertriglyceridemia and lipotoxicity have been implicated in mitochondrial dysfunction, oxidative stress, and impaired neuronal energy homeostasis, all of which contribute to progressive peripheral nerve injury [9]. Clinical studies have demonstrated that elevated serum triglycerides and dyslipidemia are independent risk factors for the development and progression of diabetic neuropathy, even after adjustment for glycemic control [10]. Beyond systemic metabolic abnormalities, diabetes is also associated with alterations in neuronal membrane lipid composition [11]. Changes in phospholipid metabolism can compromise membrane stability, disrupt axonal transport, impair signal transduction, and hinder nerve regeneration. Because neuronal membranes are highly enriched in phospholipids that are essential for maintaining structural integrity and cellular function, restoration of membrane phospholipid homeostasis has emerged as a promising therapeutic strategy [12]. Therefore, interventions capable of improving both metabolic dysregulation and neuronal membrane integrity may provide benefits that extend beyond symptomatic management and potentially slow the progression of diabetic neuropathy [13].

Choline alfoscerate (L-α-glycerylphosphorylcholine, CA) is a naturally occurring choline-containing phospholipid precursor that is metabolized into choline and glycerophosphate following absorption [14]. These metabolites serve as essential substrates for the synthesis of acetylcholine and phosphatidylcholine, respectively, thereby supporting cholinergic neurotransmission and neuronal membrane integrity [12,15]. Owing to these properties, CA has been widely used for the treatment of cognitive impairment and neurodegenerative disorders, including Alzheimer’s disease and vascular dementia [16]. Importantly, several studies have suggested that cholinergic signaling participates in the regulation of neuroinflammation and neuronal survival, processes that are critically involved in the development of diabetic neuropathy [17]. Beyond its established cognitive benefits, accumulating evidence suggests that CA possesses broader neuroprotective activities [18]. Experimental studies have demonstrated that CA enhances neuroplasticity, promotes neuronal survival, increases the expression of neurotrophic factors, and attenuates oxidative neuronal injury [18,19,20]. In addition, by facilitating phosphatidylcholine synthesis, CA may contribute to the maintenance and repair of neuronal membranes, which are frequently compromised under conditions of chronic metabolic stress [21]. Given that oxidative stress, impaired neurotrophic support, and membrane dysfunction are key contributors to the pathogenesis of diabetic peripheral neuropathy, CA may represent a promising therapeutic candidate capable of targeting multiple mechanisms involved in diabetic nerve degeneration [4]. Nevertheless, despite its established neuroprotective properties, the therapeutic potential of CA in diabetic peripheral neuropathy remains largely unexplored, and its effects on neuropathic pain, nerve degeneration, and diabetes-associated metabolic abnormalities have not been systematically evaluated [15,22].

We hypothesized that CA may exert neuroprotective effects in DPN through stabilization of neuronal membrane phospholipids, attenuation of oxidative stress, preservation of axonal integrity, and improvement of metabolic abnormalities associated with diabetic neuropathy [20]. Therefore, the aim of this study was to investigate whether early administration of CA could prevent or attenuate the development of neuropathic pain and peripheral nerve damage in an STZ-induced rat model of diabetic peripheral neuropathy. Behavioral assessments were performed to evaluate nociceptive responses, whereas histopathological analyses were conducted to assess peripheral nerve structural preservation. In addition, serum biochemical profiles were analyzed to explore potential metabolic effects associated with CA treatment. Through these investigations, we sought to determine whether CA possesses neuroprotective properties and the potential to slow the progression of diabetic peripheral neuropathy.

## 2. Results

### 2.1. Optimization of STZ Administration for the Induction of Experimental Diabetes

**Figure 1:**
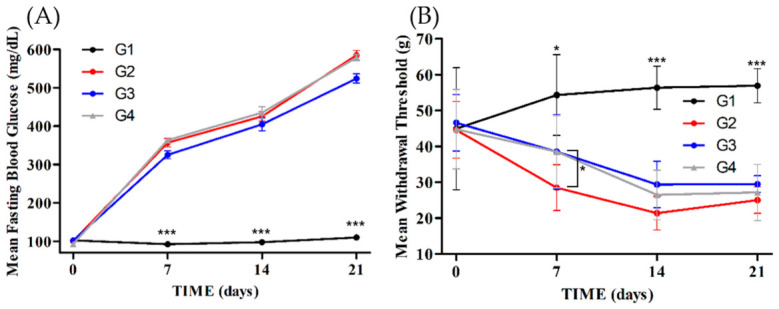
Induction of hyperglycemia and mechanical allodynia following intravenous streptozotocin (STZ) administration in male Sprague–Dawley (SD) rats. (**A**) Time-course changes in fasting blood glucose levels over 21 days after a single intravenous injection of STZ. The STZ-treated groups exhibited persistent and significant hyperglycemia throughout the experimental period compared to the normal control group (G1). (**B**) Changes in mechanical withdrawal thresholds evaluated by the von Frey filament test. STZ-treated rats developed mechanical allodynia, indicated by a significant reduction in paw withdrawal thresholds (PWTs) compared to the G1 group. Data are expressed as mean ± SD (n = 10 per group for each time point). Statistical significance was analyzed using one-way ANOVA followed by Dunnett’s post-hoc test. Asterisks (* *p* < 0.05, *** *p* < 0.001) indicate significant differences compared to the normal group (G1). This experiment was conducted using male rats. G1: Normal Control, G2: STZ 37.5 mg/kg, G3: STZ 65 mg/kg, G4: STZ 97.5 mg/kg.

**Figure A1:**
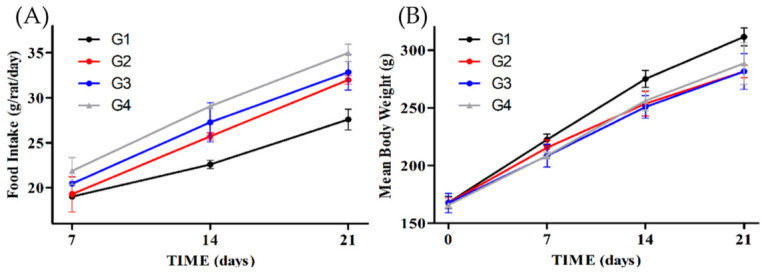
**Time-course changes in food intake and body weight over 21 days following intravenous STZ administration in male SD rats.** (**A**) Weekly monitoring of daily food intake (g/rat/day) across experimental groups. All STZ-treated diabetic groups exhibited a marked, progressive increase in food consumption over the 21-day period compared to the normal control group (G1), capturing the characteristic polyphagia of diabetes. (**B**) Progression of mean body weight changes (g) measured at scheduled timepoints. The normal control group (G1) showed a healthy and steep growth curve. In contrast, all STZ-administered groups demonstrated significantly stunted weight gain starting from day 7 despite their increased food intake, illustrating diabetic cachexia and metabolic impairment. Data are expressed as mean ± SD (n = 10 per group). G1: Normal Control, G2: STZ 37.5 mg/kg, G3: STZ 65 mg/kg, G4: STZ 97.5 mg/kg.

To establish a reproducible diabetic peripheral neuropathy model, preliminary studies were conducted to evaluate the effects of intravenous administration of streptozotocin (STZ). As shown in Figure 1, intravenous STZ administration successfully induced severe hyperglycemia; however, blood glucose levels exhibited considerable inter-animal variability and frequently exceeded the measurable range of the glucometer. In addition, animals displayed marked weight loss, deteriorating general condition, and increased mortality during the observation period. These findings suggested that although intravenous STZ administration effectively induced diabetes, the resulting phenotype was excessively severe and unsuitable for longitudinal evaluation of diabetic peripheral neuropathy and therapeutic intervention studies. Consistent with the excessive hyperglycemic phenotype, intravenous STZ administration was also associated with marked reductions in body weight, altered food intake patterns, and abnormalities in serum biochemical parameters (Appendix A Figure A1), further supporting the conclusion that this model was unsuitable for long-term therapeutic evaluation.

### 2.2. Dose Optimization of Intraperitoneal STZ Administration

**Figure 2:**
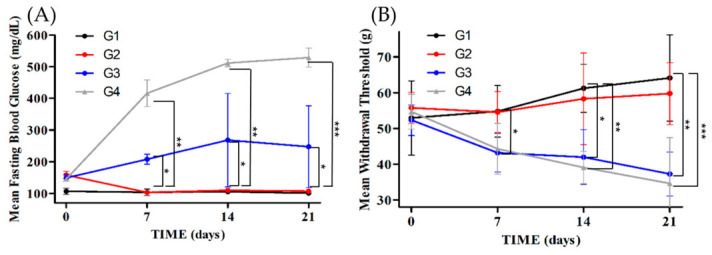
Optimization of STZ-induced diabetic peripheral neuropathy according to different doses in female SD rats. (**A**) Time-course changes in fasting blood glucose levels over 21 days following intraperitoneal administration of STZ. Hyperglycemia developed with increasing STZ doses and persisted throughout the study period. (**B**) Evaluation of mechanical sensitivity by the von Frey filament test. Reductions in paw withdrawal thresholds (PWTs) were observed, indicating the progression of mechanical allodynia. Data are expressed as mean ± SD (n = 10 per group for each time point). Statistical significance was analyzed using one-way ANOVA followed by Dunnett’s post-hoc test. Asterisks (* *p* < 0.05, ** *p* < 0.01, *** *p* < 0.001) indicate significant differences compared to the normal group (G1). This experiment was conducted using female rats. G1: Normal Control, G2: STZ 37.5 mg/kg, G3: STZ 65 mg/kg, G4: STZ 97.5 mg/kg.

**Figure A2:**
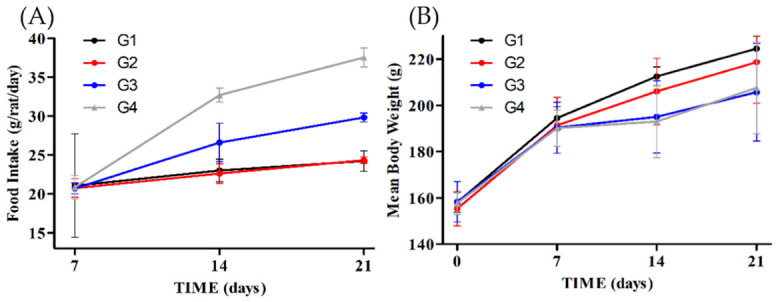
**Time-course changes in food intake and body weight over 21 days following intraperitoneal STZ administration in female SD rats.** (**A**) Weekly monitoring of daily food intake (g/rat/day) across experimental groups. STZ-treated groups exhibited an increase in food consumption, with the highest dose group (G4) demonstrating the most profound polyphagia, whereas the lowest dose group (G2) remained comparable to the normal control group (G1). (**B**) Progression of mean body weight changes (g) measured at scheduled timepoints. While the normal control (G1) and low-dose (G2) groups showed steady, healthy weight gain, the groups with successful neuropathy induction (G3 and G4) exhibited noticeably stunted body weight development starting from day 7, confirming metabolic impairment. Data are expressed as mean ± SD (n = 10 per group). G1: Normal Control, G2: STZ 37.5 mg/kg, G3: STZ 65 mg/kg, G4: STZ 97.5 mg/kg.

To establish a more stable and reproducible diabetic model, STZ was administered intraperitoneally at different dose levels. As shown in Figure 2, fasting blood glucose concentrations increased with increasing STZ doses following its treatment. Lower doses produced moderate hyperglycemia, whereas higher doses resulted in sustained elevations in blood glucose levels characteristic of diabetes mellitus. Among the tested regimens, intraperitoneal administration of STZ at 65 mg/kg consistently induced hyperglycemia while maintaining acceptable survival and general health status. Therefore, this protocol was selected for subsequent efficacy studies evaluating the effects of CA and pregabalin in diabetic peripheral neuropathy. Body weight and food intake data obtained during dose-optimization studies are presented in Appendix A Figure A2. These findings further supported the selection of the 65 mg/kg intraperitoneal STZ protocol as a reproducible model that produced sustained hyperglycemia while maintaining acceptable physiological status.

### 2.3. Establishment of the Final STZ-Induced Diabetic Peripheral Neuropathy Model

**Figure 3:**
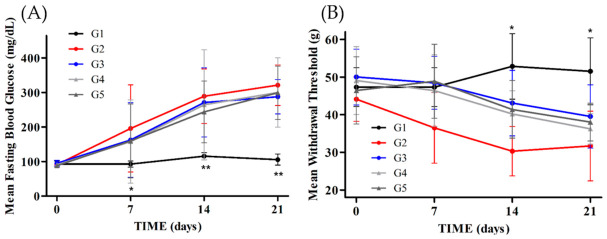
Effects of choline alfoscerate on hyperglycemia and mechanical allodynia in STZ-induced diabetic female SD rats. (**A**) Time-course changes in fasting blood glucose levels over 21 days. The STZ-induced diabetic groups (G2–G5) maintained significantly elevated glucose concentrations compared to the normal control group (G1), and treatments did not significantly alter hyperglycemia. (**B**) Changes in mechanical withdrawal thresholds evaluated by the von Frey filament test. Pregabalin (G3) and choline alfoscerate (G4 and G5) treatments significantly attenuated the reduction in paw withdrawal thresholds (PWTs) induced by STZ administration, indicating the alleviation of mechanical hypersensitivity. Data are expressed as mean ± SD (n = 10 per group for each time point). Statistical significance was analyzed using one-way ANOVA followed by Dunnett’s post-hoc test. Asterisks (* *p* < 0.05, ** *p* < 0.01) indicate significant differences compared to the normal group (G1). This experiment was conducted using female rats. G1: Normal control, G2: Negative control, G3: Pregabalin (30 mg/kg), G4: Choline alfoscerate (200 mg/kg), G5: Choline alfoscerate (300 mg/kg).

**Figure A3:**
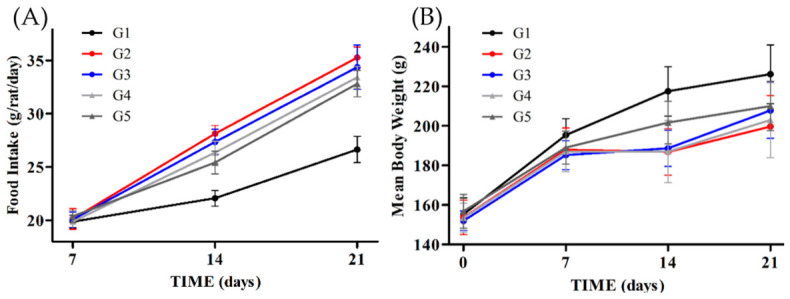
**Time-course changes in food intake and body weight over 21 days in STZ-induced diabetic female SD rats under pharmacological treatments.** (**A**) Weekly monitoring of daily food intake (g/rat/day) across experimental groups. All STZ-administered groups (G2–G5) showed a sharp, parallel increase in food consumption compared to the normal control group (G1), confirming that neither Pregabalin nor Choline alfoscerate treatment significantly affected the polyphagia induced by diabetes. (**B**) Progression of mean body weight changes (g) measured at scheduled timepoints. The normal control group (G1) displayed steady and healthy growth. Conversely, the negative control group (G2) and all drug treatment groups (G3–G5) exhibited severely stunted body weight development over the 21 days due to diabetic metabolic dysfunction, demonstrating that the treatments did not alleviate the loss of body weight despite their neuroprotective effects. Data are expressed as mean ± SD (n = 10 per group). G1: Normal control, G2: Negative control, G3: Pregabalin (30 mg/kg), G4: Choline alfoscerate (200 mg/kg), G5: Choline alfoscerate (300 mg/kg).

Based on the optimization studies described above, diabetic peripheral neuropathy was induced using a single intraperitoneal injection of STZ (65 mg/kg). As shown in Figure 3A, fasting blood glucose levels remained significantly elevated in all STZ-treated groups throughout the experimental period compared with the normal control group (*p* < 0.05). No significant differences in blood glucose concentrations were observed among the diabetic groups, including those receiving pregabalin or CA treatment. These findings confirmed successful induction and maintenance of diabetes throughout the study period and provided a stable platform for evaluating the neuroprotective effects of CA and its ability to attenuate mechanical hypersensitivity. Changes in body weight and food intake during the treatment period are presented in Appendix A Figure A3.

### 2.4. CA Attenuates Mechanical Allodynia in STZ-Induced Diabetic Rats

Mechanical sensitivity was evaluated by measuring paw withdrawal thresholds (PWTs) using the von Frey test. STZ-treated diabetic rats exhibited a marked reduction in PWT compared with normal control animals, confirming the development of mechanical allodynia [16]. As shown in Figure 3B, significant differences among the experimental groups were already evident during the first week following treatment initiation. Both pregabalin and choline alfoscerate increased PWT values compared with diabetic controls, with the greatest treatment-associated differences observed at Week 1. Although PWT values remained higher in the treatment groups than in diabetic controls throughout the study period, the magnitude of the differences gradually decreased over time. Nevertheless, the CA 300 mg/kg group consistently exhibited higher PWT values than the diabetic control group and demonstrated responses comparable to those observed in the pregabalin-treated group. Despite persistent hyperglycemia in all STZ-treated groups, CA administration significantly improved paw withdrawal thresholds compared with diabetic controls. No significant differences in blood glucose concentrations were observed between CA-treated and untreated diabetic animals throughout the experimental period.

### 2.5. CA Improves Diabetes-Associated Dyslipidemia

**Figure 4:**
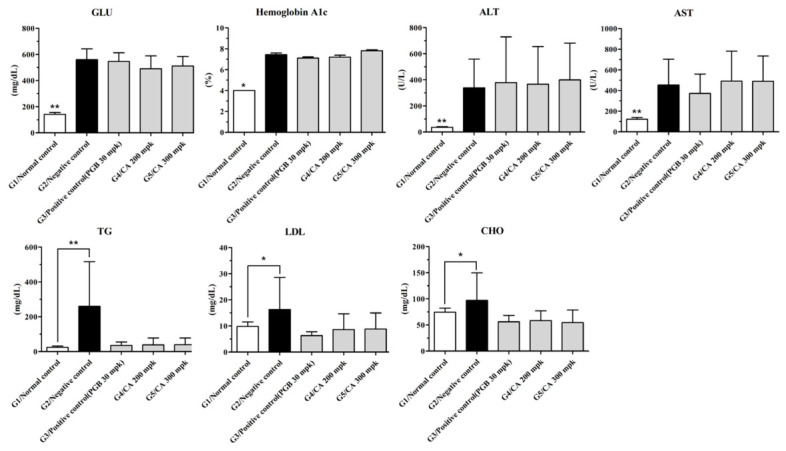
Serum biochemical profiles of STZ-induced diabetic rats on day 21 prior to necropsy. Serum levels of glucose (GLU), hemoglobin A1c (HbA1c), alanine aminotransferase (ALT), aspartate aminotransferase (AST), triglycerides (TG), low-density lipoprotein cholesterol (LDL), and total cholesterol (CHO) were measured from blood samples collected at study termination prior to necropsy. STZ administration (G2–G5) induced marked elevations in glycemic and hepatic indicators compared to the normal control group (G1). Treatment groups showed variations in lipid parameters (TG, LDL, CHO) compared to the negative control group (G2). This experiment was conducted using female rats. Statistical significance was analyzed using one-way ANOVA followed by Dunnett’s multiple comparison test. * *p* < 0.05, ** *p* < 0.01 versus the normal control group (G1). G1: Normal control, G2: Negative control, G3: Pregabalin (30 mg/kg), G4: CA (200 mg/kg), G5: CA (300 mg/kg).

Serum biochemical analyses were performed at the end of the experimental period to evaluate metabolic alterations associated with diabetes and treatment. As summarized in Table 1 and Figure 4, STZ-induced diabetic animals markedly increased serum triglyceride concentrations, whereas changes in other biochemical parameters were less consistent. Among the evaluated lipid parameters, serum triglyceride (TG) concentrations showed the most pronounced treatment-related changes. The diabetic control group demonstrated a substantial increase in TG levels compared with the normal control group (*p* < 0.05). Treatment with CA reduced serum TG concentrations in a dose-related manner, with the CA 300 mg/kg group exhibiting significantly lower TG levels than untreated diabetic animals (*p* < 0.05). The TG values observed in the high-dose CA group approached those of the normal control group. Changes in total cholesterol, HDL cholesterol, LDL cholesterol, and other biochemical parameters were also observed; however, the magnitude of these effects was less pronounced than that observed for triglycerides.

### 2.6. CA Preserves Sciatic Nerve Structure in Diabetic Rats

**Figure 5:**
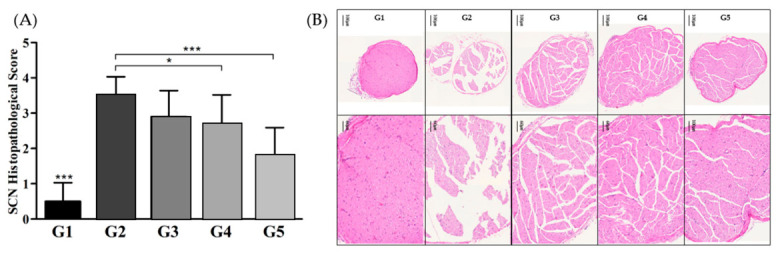
Histopathological protective effects of choline alfoscerate on the sciatic nerve in STZ-induced diabetic rats. (**A**) Histopathological scoring of sciatic nerve sections stained with H&E. Histopathological changes were evaluated using a semiquantitative scoring system (0–4) based on the severity of endoneurial edema, axonal degeneration, nerve fiber vacuolation, fascicular disorganization, myelin disruption, and overall loss of nerve architecture. Data are presented as mean ± SD (n = 10 per group). Histological scoring was performed in a randomized and blinded manner. Statistical significance was determined by one-way ANOVA followed by an appropriate post hoc test. * *p* < 0.05, *** *p* < 0.001. (**B**) Histopathological protective effects of choline alfoscerate on the sciatic nerve in STZ-induced diabetic rats. Cross-sections of the sciatic nerve were stained with Hematoxylin and Eosin (H&E). Upper panels show low-magnification views (scale bar = 100 μm), and lower panels represent higher-magnification views (scale bar = 60 μm) of the corresponding groups (n = 10 per group). This experiment was conducted using female rats. Statistical significance was analyzed using one-way ANOVA followed by Dunnett’s multiple comparison test. * *p* < 0.05, ** *p* < 0.01, *** *p* < 0.001 versus the normal control group (G1). G1 (Normal control): Shows a normal and well-preserved histological architecture of the nerve fascicle with densely packaged axons and myelin sheaths, free of endoneurial edema. G2 (Negative control): Exhibits profound structural collapse, severe endoneurial edema, and prominent nerve fiber loss with widespread vacuolation, confirming severe diabetic peripheral nerve damage induced by STZ. G3 (Pregabalin, 30 mg/kg): Demonstrates moderate degenerative changes; although tissue fissures and endoneurial separation persist, the structural fragmentation is slightly alleviated compared to G2. G4 (Choline alfoscerate, 200 mg/kg): Shows partial attenuation of nerve bundle matrix disruption, displaying a reduction in heavy tissue splitting compared to the negative control group. G5 (Choline alfoscerate, 300 mg/kg): Reveals a marked dose-related preservation of the nerve architecture. The endoneurial fibers are densely arranged with minimized structural fissures and edema, showing a morphology closest to the normal control group (G1).

Histopathological examination of sciatic nerve tissues revealed marked structural alterations in STZ-induced diabetic animals (Figure 5). Compared with the normal control group, diabetic control rats exhibited prominent nerve fiber degeneration, endoneurial edema, vacuolation, and disruption of normal fascicular organization.

Treatment with pregabalin partially reduced these pathological changes; however, abnormalities in nerve architecture remained evident. In contrast, CA treatment preserved sciatic nerve morphology in a dose-related manner. Animals receiving CA exhibited reduced vacuolation, improved fascicular organization, and decreased edema-like changes compared with untreated diabetic controls. The protective effects were most pronounced in the CA 300 mg/kg group, which displayed densely organized nerve fibers and overall histological features more closely resembling those observed in the normal control group. These findings indicate that CA attenuated STZ-induced structural damage in peripheral nerve tissues.

## 3. Discussion

The present study demonstrates that CA attenuates mechanical hypersensitivity and exerts neuroprotective effects in a streptozotocin (STZ)-induced rat model of diabetic peripheral neuropathy (DPN). Importantly, CA treatment was initiated concurrently with STZ administration, indicating that the observed benefits primarily reflect prevention or attenuation of neuropathic development rather than reversal of established nerve damage. These findings suggest that CA may possess the potential to delay neuropathic progression during the early stages of diabetes [23,24].

Diabetic peripheral neuropathy is among the most common and debilitating complications of diabetes mellitus. Despite advances in understanding DPN pathogenesis and diabetes management, current pharmacological therapies primarily focus on symptomatic pain relief, and no approved treatment has demonstrated robust disease-modifying efficacy in clinical practice [5,24,25]. Agents such as pregabalin, duloxetine, and gabapentin effectively reduce mechanical hypersensitivity [5]; however, they do not directly prevent progressive axonal degeneration or restore damaged peripheral nerves [25]. Consequently, there remains a substantial unmet clinical need for therapies capable of modifying disease progression rather than merely suppressing symptoms.

In the present study, STZ administration produced persistent hyperglycemia and mechanical allodynia, confirming successful establishment of a diabetic neuropathy model. Notably, CA treatment did not significantly alter blood glucose concentrations compared with diabetic controls, indicating that its beneficial effects were not attributable to improvements in glycemic control [6]. Despite persistent hyperglycemia, CA-treated animals exhibited improved mechanical sensitivity and preservation of peripheral nerve structure, suggesting that CA exerts its protective effects through mechanisms independent of glucose lowering [26]. One of the most important findings of this study was the preservation of sciatic nerve architecture following CA treatment. Histopathological examination revealed that diabetic control animals exhibited characteristic pathological features of DPN, including endoneurial edema, vacuolation, nerve fiber degeneration, and disruption of fascicular organization. In contrast, CA treatment reduced these abnormalities particularly in the high-dose CA group and preserved overall nerve morphology. The high-dose CA group displayed nerve structures that closely resembled those of normal controls. These observations suggest that CA exerts direct protective effects on peripheral nerve tissues and may attenuate structural degeneration associated with diabetic neuropathy [14,19,21].

An important distinction between CA and conventional mechanical hypersensitivity medications emerged from the present findings. Although pregabalin effectively improved mechanical sensitivity, its effects on nerve structure appeared limited compared with those of CA. In contrast, CA provided both functional and structural benefits, as evidenced by improvements in paw withdrawal thresholds together with preservation of sciatic nerve architecture [5,25]. These findings suggest that CA may influence pathogenic processes underlying diabetic neuropathy rather than simply improving pain-related behavior. Such dual actions suggest that CA may contribute to slowing neuropathic progression beyond conventional symptomatic analgesia [14].

Another notable observation was that the beneficial effects of CA on mechanical allodynia were most pronounced during the early phase of the experimental period [27]. Differences in paw withdrawal thresholds between treated and untreated diabetic animals were greatest during the first week and gradually diminished thereafter, although significant improvements remained evident throughout the study. This temporal pattern provides important insight into the potential mechanism of action of CA. One possible explanation is that persistent hyperglycemia may have continuously exacerbated oxidative stress, mitochondrial dysfunction, and peripheral nerve injury throughout the experimental period. Because CA did not significantly reduce blood glucose concentrations, ongoing diabetic conditions may have progressively counteracted its protective effects. In this context, CA may be particularly effective during the early stages of neuropathic development, when functional abnormalities predominate and structural nerve damage remains partially reversible [24].

Alternatively, the gradual reduction in treatment responsiveness may reflect the transition from early functional impairment to more advanced structural degeneration. During the early phase of diabetic neuropathy, neuronal dysfunction may occur before extensive axonal loss and myelin disruption. As neuropathy progresses, irreversible structural damage may accumulate, limiting the extent of functional recovery achievable by therapeutic intervention. This interpretation is supported by the observation that histopathological protection remained evident despite attenuation of behavioral differences at later time points. Together, these findings suggest that early intervention may be critical for maximizing the therapeutic efficacy of CA in diabetic neuropathy. The mechanisms underlying the neuroprotective effects of CA are likely multifactorial. CA is metabolized into choline and glycerophosphate, which serve as precursors for acetylcholine and phosphatidylcholine synthesis, respectively. Phosphatidylcholine is a major structural component of neuronal membranes and myelin sheaths, and maintenance of phospholipid homeostasis is essential for axonal integrity, membrane stability, and neuronal survival. Under diabetic conditions, oxidative stress and metabolic dysregulation accelerate membrane damage and impair nerve regeneration [14,19,21]. By supplying substrates required for membrane synthesis and repair, CA may facilitate stabilization of peripheral nerve structures and reduce susceptibility to diabetes-induced degeneration [27].

In addition to its role in membrane maintenance, CA may influence cholinergic signaling pathways. Increasing evidence indicates that cholinergic neurotransmission modulates neuroinflammatory responses, neuronal survival, and tissue repair processes within both the central and peripheral nervous systems [17,19]. Although cholinergic activity was not directly assessed in the present study, enhanced acetylcholine availability may have contributed to the protective effects observed following CA administration. Future studies investigating inflammatory mediators, neurotrophic factors, and cholinergic signaling pathways will be valuable for clarifying these mechanisms.

Interestingly, the present findings are generally consistent with previous studies investigating other choline-containing phospholipid precursors. Citicoline (CDP-choline), which shares metabolic and biochemical similarities with CA, has been reported to improve neuropathic manifestations in several experimental models, including compressive neuropathy, oxaliplatin-induced peripheral neuropathy, and streptozotocin-induced diabetic neuropathy. These beneficial effects have been attributed to enhanced phospholipid synthesis, stabilization of neuronal membranes, promotion of axonal repair, and modulation of cholinergic neurotransmission. Although CA and citicoline differ in their pharmacokinetic properties and metabolic pathways, both compounds ultimately provide choline required for phosphatidylcholine biosynthesis and neuronal membrane maintenance. Collectively, these findings extend previous observations on citicoline and provide additional evidence supporting the neuroprotective potential of CA in diabetic neuropathy [28,29,30,31].

Another noteworthy finding was the marked reduction in serum triglyceride concentrations following CA treatment. Because STZ-induced diabetic models are known to develop diabetes-associated dyslipidemia, including elevated serum triglyceride and cholesterol levels, the lipid-lowering effect observed in the present study may have contributed to the neuroprotective activity of CA [32,33,34]. Emerging evidence indicates that dyslipidemia plays an important role in the pathogenesis of diabetic neuropathy. Elevated triglycerides contribute to peripheral nerve injury through multiple mechanisms, including lipotoxicity, oxidative stress, mitochondrial dysfunction, Schwann cell damage, endothelial injury, and microvascular impairment. Recent studies have further suggested that triglyceride-rich lipid accumulation may promote neuropathy progression independently of hyperglycemia [8,10]. Therefore, the improvement in triglyceride metabolism observed in CA-treated animals may represent an additional mechanism underlying its neuroprotective effects and may partially explain the preservation of peripheral nerve structure observed in the present study. Importantly, these metabolic improvements occurred without significant reductions in blood glucose levels. This observation further supports the hypothesis that CA acts through neurobiological and metabolic pathways independent of glycemic regulation. Such effects may be particularly relevant in patients with long-standing diabetes who continue to experience neuropathy despite adequate glycemic control. The ability to improve neuropathic symptoms and preserve nerve structure independently of blood glucose reduction may therefore provide important therapeutic advantages. The optimization process was further supported by body weight, food intake, and biochemical data obtained during preliminary studies (Appendix A Figure A1 and Figure A2). These parameters provided additional evidence that excessively severe diabetic conditions were associated with substantial metabolic disturbances and reduced suitability for long-term therapeutic investigations, whereas the selected protocol maintained a more stable physiological condition while preserving the neuropathic phenotype.

Several limitations should be acknowledged. First, nerve conduction velocity and electrophysiological parameters were not evaluated. Second, molecular markers associated with oxidative stress, inflammation, apoptosis, and neurotrophic signaling were not examined. Third, ultrastructural analyses using transmission electron microscopy were not performed. Finally, because treatment was initiated simultaneously with diabetes induction, the present study primarily evaluated preventive and progression-delaying effects rather than therapeutic reversal of established neuropathy. Future studies incorporating electrophysiological assessments, molecular analyses, delayed-treatment paradigms, and longer observation periods will provide additional insight into the therapeutic potential of CA in established diabetic neuropathy.

Taken together, the present findings support a model in which choline alfoscerate attenuates diabetic neuropathy through complementary mechanisms involving preservation of peripheral nerve structure and partial correction of diabetes-associated dyslipidemia. Despite having no significant effect on blood glucose concentrations, CA attenuated mechanical hypersensitivity, preserved sciatic nerve architecture, and reduced serum triglyceride levels in STZ-induced diabetic rats. These findings suggest that the beneficial effects of CA are mediated through neuroprotective and metabolic pathways independent of glycemic control. Rather than solely improving neuropathic manifestations, CA demonstrated complementary functional, metabolic, and structural benefits under diabetic conditions. Therefore, CA may represent a promising therapeutic candidate for preserving peripheral nerve integrity and slowing neuropathic progression in diabetic peripheral neuropathy, although further mechanistic and long-term studies are required to confirm its therapeutic potential and disease-modifying effects [18,22].

## 4. Materials and Methods

### 4.1. Animals and Experimental Design

Specific pathogen-free (SPF) Sprague–Dawley (SD) rats (5 weeks of age; males, approximately 135 g; females, approximately 120 g) were obtained from Orient Bio Inc. (Seongnam, Republic of Korea), an accredited laboratory animal supplier and housed under controlled environmental conditions (23 ± 3 °C, 50 ± 10% relative humidity, and a 12 h light/dark cycle) with ad libitum access to standard laboratory chow (Teklad Global 18% Protein Rodent Diet; Envigo, Indianapolis, IN, USA) and water. Animals were acclimated for at least 7 days before the initiation of the experiments.

To establish and validate a streptozotocin (STZ)-induced diabetic peripheral neuropathy (DPN) model, three independent experiments were conducted. Male rats were used during the initial model optimization phase because of their higher susceptibility to STZ-induced diabetes, whereas female rats were used in the subsequent validation and efficacy studies to evaluate treatment responses under a more stable diabetic condition. Because the optimization and efficacy studies were conducted independently, direct sex-based comparisons were not performed and were beyond the scope of the present study. Animals were randomly assigned to experimental groups and monitored daily for clinical signs, food and water intake, body weight changes, and overall health status throughout the study.

The experimental design included a normal control group, a diabetic control group, and treatment groups receiving choline alfoscerate (CA) (Tokyo Chemical Industry Co., Ltd., Tokyo, Japan) or pregabalin (Sigma-Aldrich, St. Louis, MO, USA) following confirmation of diabetes induction. Behavioral assessments, serum biochemical analyses, and histopathological evaluations were performed according to the study schedule described in the Methods section. All animal procedures were conducted in accordance with the Guide for the Care and Use of Laboratory Animals and complied with institutional ethical guidelines. The study protocol was reviewed and approved by the Institutional Animal Care and Use Committee (IACUC) of Korea University (Approval No. KUIACUC-2025-0056).

### 4.2. Induction of Diabetes and Experimental Groups

Diabetes mellitus was induced using streptozotocin (STZ; Sigma-Aldrich, St. Louis, MO, USA). Preliminary optimization studies were conducted to establish a reproducible diabetic peripheral neuropathy (DPN) model. Intravenous administration of STZ via the tail vein was initially evaluated; however, this approach resulted in excessive hyperglycemia, increased mortality, and considerable inter-animal variability. Therefore, the final experimental model was established using a single intraperitoneal (IP) injection of STZ at a dose of 65 mg/kg dissolved in freshly prepared citrate buffer (0.1 M, pH 4.5; Sigma-Aldrich, St. Louis, MO, USA). Seventy-two hours after STZ administration, fasting blood glucose levels were measured using tail vein blood samples. Animals with fasting blood glucose concentrations ≥300 mg/dL were considered diabetic and included in subsequent experiments. Diabetic animals were randomly assigned to treatment groups following confirmation of hyperglycemia.

The animals were allocated to five experimental groups: (G1) normal control, (G2) diabetic control, (G3) pregabalin-treated diabetic group (30 mg/kg), (G4) CA-treated diabetic group (200 mg/kg), and (G5) CA-treated diabetic group (300 mg/kg). Pregabalin was included as a positive control because of its well-established efficacy in alleviating mechanical hypersensitivity in experimental diabetic neuropathy models [15]. Pregabalin and CA were administered intraperitoneally once daily beginning on the day of STZ injection and continued throughout the 21-day experimental period. This dosing schedule was selected to evaluate the ability of CA to prevent or attenuate the development of diabetic neuropathy rather than to reverse established nerve damage. This treatment regimen was designed to evaluate the potential preventive and disease-modifying effects of CA during the development of diabetic peripheral neuropathy. Animals in the normal and diabetic control groups received an equivalent volume of vehicle according to the same dosing schedule. Behavioral assessments were performed throughout the treatment period to evaluate mechanical hypersensitivity responses. At the end of the study, blood samples and sciatic nerve tissues were collected for biochemical and histopathological analyses. All behavioral, biochemical, and histopathological evaluations were conducted by investigators blinded to group allocation.

### 4.3. Blood Glucose Measurement

Fasting blood glucose levels were measured weekly throughout the experimental period following an overnight fast (8–12 h). Blood samples were collected from the tail vein using sterile lancets, and glucose concentrations were determined using a portable glucometer (Accu-Chek Performa, Roche Diagnostics, Mannheim, Germany). Blood glucose measurements were performed to confirm successful induction of diabetes and to monitor maintenance of the diabetic state throughout the study.

### 4.4. Assessment of Mechanical Allodynia

Mechanical sensitivity was evaluated using a Dynamic Plantar Aesthesiometer (Ugo Basile, Gemonio, Italy). Prior to testing, animals were individually placed in transparent acrylic chambers positioned on an elevated wire mesh platform and allowed to acclimate for 20 min. Mechanical stimuli were applied to the plantar surface of the hind paw using a blunt metal filament (0.5 mm in diameter). The applied force increased automatically from 0 to 50 g over a period of 20 s.

The paw withdrawal threshold (PWT) was automatically recorded as the force (g) required to elicit a withdrawal response. To prevent tissue injury, a cut-off force of 50 g was imposed. Each hind paw was tested at least three times with a minimum interval of 3 min between consecutive measurements, and the mean value was used for statistical analysis. Measurements were obtained from both hind paws and averaged for each animal. All behavioral assessments were performed by an investigator blinded to the treatment allocation. Testing was performed at the same time of day to minimize circadian variation.

### 4.5. Serum Biochemical Analysis

At the end of the experimental period, animals were deeply anesthetized with isoflurane (Hana Pharm Co., Ltd., Seoul, Republic of Korea) and euthanized by exsanguination, and blood samples were collected for biochemical analyses. Blood was transferred into serum separator tubes (SST; BD Biosciences, Franklin Lakes, NJ, USA), allowed to clot at room temperature, and centrifuged at 1500× *g* for 15 min. The resulting serum was collected and stored at −80 °C until analysis. Serum biochemical parameters were measured using an automated clinical chemistry analyzer (Hitachi 7180; Hitachi High-Technologies Corporation, Tokyo, Japan). To assess the systemic safety of the treatments, serum liver function markers, including aspartate aminotransferase (AST) and alanine aminotransferase (ALT), were measured.

To investigate metabolic alterations associated with diabetes and diabetic peripheral neuropathy, serum lipid profiles were also assessed, including total cholesterol (CHO), high-density lipoprotein cholesterol (HDL), low-density lipoprotein cholesterol (LDL), and triglycerides (TG). In addition, glycemic status was evaluated by measuring serum glucose and glycated hemoglobin (HbA1c) concentrations. These analyses were performed to characterize the metabolic environment associated with diabetic neuropathy and to determine whether CA treatment influenced diabetes-related metabolic disturbances.

### 4.6. Histopathological Examination

At the end of the experimental period, sciatic nerve tissues were carefully dissected and fixed in 10% neutral-buffered formalin. Following fixation, tissues were routinely processed, embedded in paraffin, and sectioned at a thickness of 4–5 μm. Tissue sections were stained with hematoxylin and eosin (H&E) according to standard histological procedures.

Histopathological examination was performed using light microscopy to evaluate structural alterations associated with diabetic peripheral neuropathy, including endoneurial edema, nerve fiber degeneration, vacuolation, fascicular disorganization, and overall loss of nerve architecture. For histopathological evaluation, a scoring system based on predefined assessment criteria was established (Table 2). Histological changes were graded on a five-point scale ranging from 0 to 4. To minimize potential bias, tissue sections from all experimental groups were randomized prior to evaluation. Representative images were acquired using a digital microscope imaging system at low and high magnifications under identical imaging conditions for all groups. Histological assessments were performed by an investigator blinded to treatment allocation. Comparative evaluation of histopathological alterations was performed among the experimental groups to assess the protective effects of CA on peripheral nerve morphology.

### 4.7. Statistical Analysis

All data are presented as mean ± standard deviation (SD). Statistical analyses were performed using GraphPad Prism version 5.0 (GraphPad Software, San Diego, CA, USA). For endpoint measurements, including serum biochemical parameters, differences among groups were analyzed using one-way analysis of variance (ANOVA) followed by Dunnett’s multiple comparison test, with the diabetic control group serving as the reference group. For repeated measurements, including blood glucose levels, body weight, and mechanical sensitivity assessments, two-way repeated-measures ANOVA was used to evaluate the effects of treatment and time, followed by Dunnett’s multiple comparison test when appropriate. A value of *p* < 0.05 was considered statistically significant.

Animals were randomly assigned to experimental groups following confirmation of diabetes induction. Behavioral assessments, histopathological evaluations, and data analyses were conducted by investigators blinded to treatment allocation to minimize potential bias. Sample sizes were determined based on previous studies employing STZ-induced diabetic peripheral neuropathy models and on the expected treatment-related differences in mechanical allodynia outcomes. The selected group sizes were considered sufficient to detect biologically relevant effects while minimizing animal use.

## Figures and Tables

**Table 1:** Serum biochemical and metabolic parameters measured at study termination.

Group		Bio_Chemistry						
		Glu (mg/dL)	HbA1c (%)	ALT (U/L)	AST (U/L)	TG (mg/dL)	LDL-C (mg/dL)	CHO (mg/dL)
G1 Normal control	Mean	141.80	4.00	36.60	122.10	24.70	9.80	74.50
	S.D.	13.91	0.00	5.35	16.84	7.46	1.78	7.84
G2 Negative control	Mean	560.90 ***	7.44 ***	339.20 *	454.00 **	260.10 ***	16.30 *	97.10
	S.D.	82.62	0.16	219.54	248.74	256.86	12.27	52.57
G3 Pregabalin 30 mg/kg	Mean	546.60 ***	7.11 ***/###	379.20 **	372.10 *	35.60 ###	6.30 ##	56.00 ##
	S.D.	66.51	0.12	350.47	187.89	19.75	1.49	12.47
G4 Choline alfoscerate 200 mg/kg	Mean	491.30 ***/#	7.20 ***/###	367.20 **	491.50 ***	38.60 ###	8.60 #	58.40 ##
	S.D.	97.56	0.19	288.35	290.15	39.80	6.09	18.71
G5 Choline alfoscerate 300 mg/kg	Mean	511.80 ***	7.80 ***/###	399.70 **	488.50 ***	39.70 ###	8.80 #	54.60 ##
	S.D.	73.34	0.11	281.63	246.49	38.42	6.18	24.20

Values are presented as mean ± SD (n = 10 per group). This experiment was conducted using female rats. Statistical significance was analyzed using one-way ANOVA followed by Dunnett’s multiple comparison test. * *p* < 0.05, ** *p* < 0.01, *** *p* < 0.001 versus the normal control group (G1); # *p* < 0.05, ## *p* < 0.01, ### *p* < 0.001 versus the diabetic control group (G2).

**Table 2:** Histopathological scoring criteria for sciatic nerve (SCN) damage in hematoxylin and eosin (H&E) staining.

Score	Grade
0	Normal architecture
1	Minimal endoneurial edema and focal vacuolation
2	Moderate edema and focal axonal degeneration
3	Marked degeneration and fascicular disorganization
4	Severe structural collapse and widespread fiber loss

## Data Availability

The original contributions presented in the study are included in the article, further inquiries can be directed to the corresponding author.
